# Determinants of Weight Status and Body, Health and Life Satisfaction in Young Adults

**DOI:** 10.3390/nu16101484

**Published:** 2024-05-14

**Authors:** Julia Suwalska, Sylwia Łukasik, Maciej Cymerys, Aleksandra Suwalska, Paweł Bogdański

**Affiliations:** 1 Department of Treatment of Obesity, Metabolic Disorders and Clinical Dietetics, Poznan University of Medical Sciences, 60-569 Poznan, Poland; 2 Institute of Human Biology and Evolution, Faculty of Biology, Adam Mickiewicz University in Poznan, 61-614 Poznan, Poland; 3 Department of Internal Medicine, Poznan University of Medical Sciences, 60-786 Poznan, Poland; 4 Department of Mental Health, Chair of Psychiatry, Poznan University of Medical Sciences, 60-572 Poznan, Poland

**Keywords:** emotional eating, obesity, young adults, depression, physical activity, life satisfaction

## Abstract

Health behaviors include behavioral patterns and habits that relate to health maintenance, restoration and improvement. They do not only affect the physical condition; they are also associated with life satisfaction. In our study, we focused on young adulthood, a specific lifespan period for establishing long-term health behavior patterns. The aim of the present study was to investigate depressive symptoms, lifestyle and eating behaviors and delineate their associations with overweight/obesity and body, health and life satisfaction in young adults in Poland. We enrolled 800 students (81.4% females and 18.6% males). Diet, physical activity, depressive symptoms, eating behaviors and body, health and life satisfaction were assessed. Multivariate logistic regression models were employed. Almost half of the participants in our study had at least mild symptoms of depression. Symptoms of depression significantly reduced the odds of satisfaction with body, health and life, whereas physical activity increased them. Overweight/obesity significantly reduced the odds of body and health satisfaction. In women, a history of depression and emotional eating increased the odds of being overweight/obese. The results of our study may contribute to the development of educational programs and intervention strategies for young adults.

## 1. Introduction

A quarter of a century ago, the World Health Organization (WHO) described a healthy lifestyle as a way of living that lowers the risk of being seriously ill or dying early [1]. According to this definition, a healthy lifestyle means engaging in regular physical activity, refraining from smoking, limiting alcohol consumption, and eating healthily to prevent being overweight. These behaviors contribute to better physical health and foster mental well-being [1,2]. A healthy lifestyle is further defined as a combination of lifestyle components associated with lower mortality and better health outcomes [3]. Health behaviors include behavioral patterns and habits that relate to health maintenance, health restoration and health improvement [4,5].

Most studies on lifestyle investigate smoking, diet, physical activity and overweight or obesity, and some also take alcohol use into account [3]. It has been found that smoking, physical inactivity, an unhealthy diet, obesity and other lifestyle behaviors are associated with the development of diseases such as cancer, heart disease, stroke and diabetes [6]. Individuals with four or more healthy behaviors have a 66% reduced risk of all-cause mortality [7] compared to those with an unhealthy lifestyle.

Health behaviors do not only affect the physical condition; they are also associated with subjective well-being (SWB) [8]. SWB comprises life satisfaction and the balance between positive and negative affect [9]. Life satisfaction reflects the extent to which various aspects of an individual’s life are considered satisfying or fulfilling. It can be evaluated globally or divided into various domains, including, among others, satisfaction with self, health, work, family and leisure [10]. College students rate life satisfaction as very important [11]. In youth, body satisfaction may protect against excessive weight gain and eating disorders [12,13].

It is particularly important to investigate health behaviors in young adults (18–25 years of age) [14]. This is a period in life when young people experience dynamic and complex changes and set themselves on the path to adulthood, change their living arrangements, end their education, start working and change their primary relationships [15,16,17]. This is the time of transition from parental supervision to individual responsibility [13], from adolescent/dependent roles to adult/independent roles [17]. These life transitions may affect health behaviors [18]. During the shift from adolescence into young adulthood, a decline in physical activity and dietary habits is observed [15,19,20]. Young adulthood is often associated with poor lifestyle behaviors [21]. Unhealthy behaviors tend to continue into middle and late adulthood, predisposing individuals to preventable chronic conditions such as cardiovascular and respiratory diseases and diabetes [22].

An analysis of data from over 24,000 adults aged 18–24 years from the Behavioral Risk Factor Surveillance System (BRFSS) showed that almost half of them had at least one chronic condition. The most prevalent conditions were obesity (25.5%) and depression (21.3%) [23]. An increase in weight in young people may be attributed to a poor diet, excessive alcohol intake and low levels of physical activity [24]. Compared to other age groups, young adults are more likely to manage stress by engaging in unhealthy and weight-promoting behaviors, such as excessive eating and drinking alcohol [25]. Young adulthood represents a critical period for weight control, as the rate of weight gain is greatest at this time of life [25].

Young adults are also exposed to a high risk of developing mental health problems, as the onset of 75% of lifetime mental disorders occurs before the age of 25 [26]. The beginning of higher education coincides with the age of onset of problem behaviors (e.g., alcohol abuse and internet addiction) and mental health problems (e.g., depression and anxiety) [27]. One in five university students experiences mental health concerns, a five-fold increase over the past decade [28]. Mental disorders like depression and anxiety may negatively affect global and domain-specific life satisfaction [29,30].

The aim of this study was to investigate depressive symptoms, lifestyle and eating behaviors and delineate their associations with overweight/obesity and body, health and life satisfaction in young adults in Poland.

## 2. Materials and Methods

### 2.1. Study Procedure and Participants

The study was approved by the Ethical Committee of the Poznan University of Medical Sciences on 6 September 2018 by Resolution No. 857/18. The study was designed as a cross-sectional study and conducted among undergraduate health sciences students at the Poznan University of Medical Sciences, Poland. Data were collected between October 2019 and March 2022. Questionnaires were distributed by researchers to 985 students in their 1st-3rd year of physiotherapy, midwifery, nursing, public health, emergency medicine, occupational therapy, cosmetology, or dietetics during study courses. Eight hundred and five questionnaires were returned (response rate 81.7%), and five forms were excluded due to missing data (sex, age, weight). All participants enrolled voluntarily and provided written informed consent. The final study sample (800 students) included 81.4% females and 18.6% males, with a mean age of 20.4 ± 1.7 (range 18–25 years).

### 2.2. Measures

Sociodemographic characteristics (gender, age, sex, financial situation, self-reported health data, etc.) were collected. Smoking status was assessed with a question from the European Health and Behavior Study (EHBS) [31]. Participants were classified as current smokers or non-smokers.

BMI (body mass index) was calculated as body mass (kg) divided by height squared (m^2^) from the students’ self-reported height and weight. According to the World Health Organization, participants were classified as underweight (BMI < 18.5 kg/m^2^), healthy weight (BMI 18.5 to 24.9 kg/m^2^), overweight (BMI 25.0 to 29.9 kg/m^2^), and obese (BMI 30.0 kg/m^2^ or higher) [32]. For the purpose of statistical analysis, the categories of overweight and obese were merged into one category.

Symptoms of depression were assessed with the Beck Depression Inventory (BDI) [33], a 21-item multiple-choice questionnaire. For every question, the severity of the symptom is assessed on a scale of 0 to 3, and the score is the sum of the points. The cut-off scores were as follows: 0–9, normal range; 10–18, mild-to-moderate depression; 19–29, moderate-to-severe depression; and 30–63, severe depression [34].

The Food Frequency Questionnaire (FFQ) [35] was used to collect information on the frequency of consumption of 62 product groups. Consumption frequency for each food item was indicated by choosing one of six answers: never or very rarely, once a month or less, several times a month, several times a week, daily, or a few times a day. Subsequently, the answers were converted into daily frequency and aggregated into twenty-five food categories [36]. Alcohol consumption was assessed by a question from FFQ [35] following the same conversion procedure.

The International Physical Activity Questionnaire (IPAQ) [37] was used to evaluate the level of physical activity. The short form of IPAQ asks about three specific types of activity (walking, moderate-intensity activities, and vigorous-intensity activities) undertaken in the last 7 days. Based on the results, participants were divided into three levels of physical activity (low, moderate or high). In the analyses, moderate/high activities were compared to low physical activity as a reference category.

To assess eating behaviors, a Polish validated version of the Three Factor Eating Questionnaire (TFEQ-18) was used [38,39]. This questionnaire measures three aspects of eating behaviors: restrained eating (6 items, conscious restriction of food intake in order to control body weight or to promote weight loss), uncontrolled eating (9 items, tendency to eat more than usual due to a loss of control over intake), and emotional eating (3 items, inability to resist emotional cues) [38]. Seventeen items were answered on a 4-point scale and one on an 8-point scale. The results were summated into scale scores for cognitive restraint, uncontrolled eating, and emotional eating and transformed to a 0–100 scale [40].

Body satisfaction was assessed using the question “How satisfied are you with your body appearance?”, health satisfaction using the question “How satisfied are you with your health?” and life satisfaction using the question “How satisfied are you with your life, overall?” For each item, there were five Likert response categories: (1) very dissatisfied, (2) dissatisfied, (3) neither dissatisfied nor satisfied, (4) satisfied and (5) very satisfied [41,42]. Answers were dichotomized into ‘not satisfied’ (1–3) and ‘satisfied’ (4–5).

### 2.3. Statistical Analysis

Data were analyzed using the Statistica 13 package (Statsoft, Kraków, Poland). Continuous data are presented as mean and standard deviation (SD), whereas categorical variables are expressed as frequencies and percentages. A *T*-test for continuous variables and a chi-squared test for categorical variables (with Yates’s correction when the class size did not exceed 10 items) were used. The level of significance was set at *p* < 0.05.

To address the research purposes, logistic regression models were employed. All of the analyses were also conducted separately for women and men.

First, a univariate logistic regression was conducted to assess the potential association between dependent variables, i.e., overweight/obesity, body satisfaction, health satisfaction and life satisfaction, and independent variables. The independent variables tested for their association with overweight/obesity included sociodemographic factors (sex, financial status, smoking), medical history (history of depression, hypothyroidism), eating behaviors (emotional eating, cognitive restraint, uncontrolled eating), symptoms of depression (no/yes), physical activity (low/moderate/high) and consumption of food products measured by FFQ. The independent variables tested for their association with body satisfaction, health satisfaction and life satisfaction included sociodemographic factors (sex, financial status, smoking), medical history (history of depression, hypothyroidism), eating behaviors (emotional eating, cognitive restraint, uncontrolled eating), symptoms of depression (no/yes), physical activity (low/moderate/high) and overweight/obesity.

Then, hypotheses of how different independent variables (factors) may act together to affect the dependent variables (overweight/obesity, body satisfaction, health satisfaction and life satisfaction) and the nature (positive or negative) and significance of the relationship were tested. For this purpose, variables identified as statistically significant in the univariate analysis (*p* < 0.05) were entered into the multivariate analysis.

## 3. Results

### 3.1. Characteristics of the Studied Group

The main characteristics of the participants are summarized in Table 1.

The studied group consisted of 800 students: 651 (81.4%) female and 149 (18.6%) male; mean age 20.4 ± 1.7. The participants were students in their 1st–3rd year of physiotherapy (336; 42.0%), midwifery (278; 34.8%), nursing (108; 13.5%), public health (21; 2.6%), emergency medicine (34; 4.3%), occupational therapy (17; 1.5%), cosmetology (4; 0.5%), or dietetics (2; 0.3%). In the study group, 9.3% of the participants were underweight, 71.8% had a normal weight, 14.3% were overweight and 4.8% were obese. Hypothyroidism was reported by 68 subjects (8.5%) and a history of depression by 52 (6.5%). One hundred and fifty-seven participants (19.6%) reported smoking tobacco. Of the 800, 63.3% of the participants reported body satisfaction, 57.3% reported health satisfaction and 76.2% reported life satisfaction. In terms of physical activity, 20.5% of the participants had a low level, 46.1% had a moderate level, and 33.4% had a high level (IPAQ). Almost half of the respondents (49.6%) showed symptoms of depression.

### 3.2. Comparison between Men and Women

The results are presented in Table 2.

Women significantly more often reported hypothyroidism than men. Men significantly more often showed moderate/high physical activity levels according to the IPAQ category and significantly more often reported that they smoked tobacco. They also reported a higher consumption of refined grains, butter, other fats, potatoes, processed meat, red and white meat, alcohol, sweetened beverages and juices. Women had a significantly higher severity of depressive symptoms. Furthermore, symptoms of depression were more frequently observed in women than in men (53.2% vs. 33.8%, *p* < 0.05). Women were significantly less likely to be satisfied with their body, health and life. Cognitive restraint and emotional eating were significantly more pronounced in women.

### 3.3. Overweight/Obesity

In the multivariate analysis (Table 3), male gender, cognitive restraint, emotional eating and the consumption of red meat and sweetened beverages were identified as statistically significant risk factors in predicting overweight/obesity, whereas the consumption of refined grains reduced the risk. In women, the odds of being overweight/obese were increased by hypothyroidism, a history of depression, emotional eating, and red meat consumption. After adjusting for other factors in the model, only cognitive restraint significantly increased the odds of overweight/obesity in men.

### 3.4. Body Satisfaction

Table 4 shows the results of a multivariate logistic regression analysis predicting body satisfaction. The odds of body satisfaction were significantly reduced by overweight/obesity, symptoms of depression, cognitive restraint and uncontrolled eating, whereas moderate/high physical activity significantly increased the odds. The model for women included the same significant factors. In men, only overweight/obesity and symptoms of depression remained significant after adjustment for other factors.

### 3.5. Health Satisfaction

Table 5 presents the multivariate logistic regression model for predicting health satisfaction. Hypothyroidism, history of depression, overweight/obesity, symptoms of depression measured by BDI and emotional eating reduced the odds of health satisfaction, whereas moderate/high physical activity significantly increased the odds. In women, the same factors had a significant impact. Symptoms of depression and uncontrolled eating significantly reduced the odds of health satisfaction in men.

### 3.6. Life Satisfaction

In the multivariate analysis (Table 6), the odds of life satisfaction were reduced by smoking and symptoms of depression. An average or high financial status compared to a low financial status and moderate/high physical activity compared to low physical activity increased the odds of life satisfaction. In women, the odds were reduced by symptoms of depression and increased by an average or high financial status and moderate/high physical activity. In men, the odds were significantly reduced by depressive symptoms.

## 4. Discussion

In our study, we focused on young adulthood, a specific lifespan period during which many people experience weight gain. This is also the time to establish long-term health behavior patterns [15]. As overweight and obesity are associated with many negative health consequences, including increased risk of cardiovascular diseases and cancer [43], it is important to redirect resources towards the targeted primary prevention of weight gain in early adulthood [44]. To do this, it is important to analyze risk factors such as emotional eating, poor diet or depressive symptoms, which are potential targets for intervention.

Our study also analyzed factors influencing body, health and life satisfaction in young adults. It is believed that focusing on the health and well-being of young adults is especially important as it may help them to successfully embrace adult roles as healthy citizens, workers and parents [16].

Only approximately 57% of the subjects in our study were satisfied or very satisfied with their health (men significantly more often than women: 70.7% vs. 54.3%). According to the American College Health Association (ACHA), in 2019, over eighty percent of college students described their health as ‘good’, ‘very good’ or ‘excellent’ [45]. Among Italian students, having good or very good health has been declared by 77% [46], and in the United Kingdom, 47.5% have perceived their own health as ‘very good’ or ‘excellent’ [47]. Higher health satisfaction among men is in line with other studies [46,48]. Three-quarters of the subjects in our study were satisfied with their life, this being significantly more frequent in men. The results of previous studies on gender differences as regards to life satisfaction are inconsistent and vary across countries [42,49]. Body satisfaction was observed in 63.3% of our study participants, significantly more often in men. Compared with men, women have shown lower body satisfaction in a study by Godoy-Izquierdo et al. [50].

Both an average and high financial status increase the odds of life satisfaction (2.5 times and 8.7 times, respectively). In a study by Prémusz et al. [51], financial status was the strongest predictor of life satisfaction among women, whereas in a study by He et al., women in households with a lower wealth status reported poorer subjective health, life satisfaction and happiness [52]. Similar results were obtained in a Spanish study [48].

In our study, smoking decreased the odds of life satisfaction. The results of a longitudinal study by Lappan et al. revealed that smoking predicted poorer life satisfaction, less optimism, positive affect and less purpose in life [53]. In a study by Grant et al. [42], life satisfaction was positively associated with non-smoking among students from 21 countries. The literature rarely considers decreasing life satisfaction as a negative consequence of smoking [53]. However, the relationship might be bidirectional—smoking contributes to low life satisfaction and life dissatisfaction leads to smoking [42].

### 4.1. Physical Activity

In our study, as many as 79.5% of the participants reported a moderate/high level of physical activity. In a study by Pengpid and Peltzer [41], 42.2% of the participants reported a low level of activity, 36.5% a moderate level of activity, and 21.2% a high level of physical activity. The higher level of physical activity in our study may be due to the fact that it was conducted among students of health sciences, including 42.0% of physiotherapy students who are generally engaged in sports and are more health-oriented. Regular physical activity is proven to help prevent and manage noncommunicable diseases (NCDs), such as heart disease, stroke, diabetes and obesity. It can improve mental health, quality of life and well-being [54].

We found a significantly higher level of physical activity among men, which is in line with previous studies [55,56,57]. Possible causes of these gender discrepancies include cultural standards and values, traditional roles and the lack of social and community support for women’s participation in physical activity [58]. More opportunities for safe and accessible leisure-time activity need to be provided to women in order to increase their overall levels of activity [55].

In our study group, moderate/high physical activity significantly increased the odds of body, health and life satisfaction. The results of the aforementioned study by Pengpid et al. [41] revealed that moderate/high physical activity increases the odds of higher life satisfaction, greater happiness and better perceived health, whereas longer sedentary behavior decreases them. In previous studies among students, physical activity has been associated with happiness and better perceived health [47,59]. A large European study conducted among women of reproductive age has shown that regular physical activity results in better life satisfaction [51]. Leisure-time physical exercise has also been associated with better life satisfaction in a large population of students [42]. Physical activity can and should be integrated into the settings in which people live, work and play [54]. Quality physical education and supportive environments can contribute to long-lasting healthy, active lifestyles [54]. Universities can encourage physical activity among students by offering extra physical education classes and exercise facilities and promoting walking and cycling as means of transportation and active recreation.

### 4.2. Overweight/Obesity

Overweight/obesity was observed in almost 20% of the study’s participants. Overweight/obesity significantly reduced the odds of body satisfaction (3.0 times) and health satisfaction (1.7 times). In a study by Godoy-Izquierdo et al. among Spanish adults, a higher BMI was associated with lower body satisfaction among men and women. Similar results were presented by Streeter et al. for a group of young adults in Canada [60]. Among Portuguese and Brazilian students, a higher BMI has been associated with greater dissatisfaction with general body appearance in men and body shape in women [61]. In a systematic review by Weinberger et al. [62], individuals with obesity reported higher levels of body dissatisfaction than normal-weight individuals. In a systematic review by Puhl and Heuer, obesity was shown to increase the risk of depression, low self-esteem and poor body image and was associated with stigmatizing attitudes [63].

In our study, a poorer quality of diet in men (a higher consumption of refined grains, butter and other fats, meat, sweetened beverages and alcohol) was observed. Data from other studies indicate that, compared to men, women are more likely to report eating fruits, vegetables, cereals, milk, dairy and whole-grain products and avoiding red meat, eggs, alcohol and high-sucrose foods [64,65,66,67].

We noted that the consumption of red meat and sweetened beverages increased the risk of overweight/obesity, and the intake of refined grains decreased it. Whereas the role of red meat and sweetened beverages as risk factors for weight gain is well established [68,69], our results concerning refined grains are somewhat counterintuitive since refined grain intake is widely assumed to be associated with adverse health outcomes. However, the results of 11 meta-analyses of prospective cohort studies demonstrated that refined grain intake has not been associated with all-cause mortality, type 2 diabetes, cardiovascular disease, coronary heart disease, stroke, hypertension or cancer [70]. Furthermore, white bread is included in a common Polish breakfast, and a link between skipping breakfast and overweight/obesity has been reported previously [71].

### 4.3. Depression

Almost half of the participants in our study had at least mild symptoms of depression, and 6.5% reported a history of depression. Depressive symptoms were significantly more common in women than in men. The 2019 data from the American College Health Association showed that within the previous 12 months, 20.0% of students (11.7% of men and 22.5% of women) had been diagnosed with or treated by a professional for depression [45], and 45.1% of the participants reported feeling so depressed that it was difficult to function [45]. According to Lipson et al. [72], there was a 134.6% increase in symptoms of depression among U.S. students between 2013 and 2021 (from 17.4% to 40.8%). Depressive disorders are the leading cause of years lost due to disability (YLDs) for females aged 15–24 years and affect more females than males in all young people’s age groups [73].

In our study, symptoms of depression significantly reduced the odds of satisfaction with body (6.3 times), health (2.6 times) and life (5.3 times). Moreover, a history of depression significantly reduced the odds of health satisfaction. In a longitudinal study, Fergusson et al. [74] showed a bidirectional association between mental health problems (including major depression) and life satisfaction.

In this group of women, a history of depression increased the odds of developing obesity by 2.2 times. In a longitudinal study by Konttinen et al., depression predicted higher BMI and waist circumference gain over 7 years [75]. In a systematic review and meta-analysis of longitudinal studies by Luppino et al. [76], baseline depression (symptoms and disorder) increased the odds of developing obesity. Therefore, patients with obesity should be screened for depression, and patients diagnosed with depression should have their body mass monitored [77].

### 4.4. Hypothyroidism

In our study, hypothyroidism was significantly associated with overweight/obesity. The common symptoms of hypothyroidism in adults, such as fatigue, mood impairment, weight gain, constipation and menstrual disturbance [78], are not specific and can be confused with those of obesity [79]. A higher prevalence of overt and subclinical hypothyroidism in obesity has been shown [80], but some studies suggest that changes in thyroid hormones could be the consequence, rather than the cause, of weight gain [81]. The European Society of Endocrinology Clinical Practice Guideline recommends an assessment of thyroid function in people with obesity [79].

### 4.5. Eating Behaviors

We confirmed the results of previous studies showing that emotional and restrained eating are more common in women [40,82,83]. Our results showed that higher levels of emotional eating increased the odds of overweight/obesity in women. This is in line with the results of a 5-year study by van Strien et al. [84], which showed that emotional eating predicted future body weight gain in women but not in men. Also, in a study by Anglé, higher scores of cognitive restraint and emotional eating were associated with a higher BMI [85].

In a study on a Swedish population, cognitive restraint was associated with a higher body weight in adolescents, whereas this was the case for uncontrolled eating and emotional eating in adult women [86]. High levels of emotional eating were associated with overweight in a study by Lluch et al. [83]. An association between emotional eating and BMI has also been reported in young people [87,88].

In our study, cognitive restraint was significantly associated with overweight/obesity in men. An association between cognitive restraint and overweight/obesity has been reported previously [83,89]. It should be noted, however, that in view of the fact that it is a cross-sectional study, it is possible that the dependence is the other way around: a higher body weight makes people eat in a more restrained way in order to control their weight [85]. In a French longitudinal study, restrained eating was not associated with an increase in adiposity over time, but a higher initial BMI was associated with a greater increase in cognitive restraint scores in participants [90].

In women, a higher level of emotional eating reduced the odds of health satisfaction, whereas cognitive restraint reduced the odds of body satisfaction. A higher level of uncontrolled eating reduced body satisfaction in women and health satisfaction in men. In a study by Jáuregui-Lobera et al. [89], cognitive restraint was associated with the perception of being overweight.

These results are of significance since they show that poor eating behaviors can lead to overweight and obesity and can reduce body, health and life satisfaction. This is why eating behaviors should be considered in the development of lifestyle interventions [91], for example, by teaching emotion regulation skills [92].

### 4.6. Limitations

Several limitations should be acknowledged. First, the results were obtained using self-reported questionnaires and self-reported height and weight data. The diagnosis of hypothyroidism was self-reported and was not confirmed by laboratory tests. Second, the sample is gender-imbalanced, with male respondents being in the minority, which is due to the high proportion of female students in health-related studies. This limitation was addressed by performing a separate analysis of the data for women and for men. Third, body, health and life satisfaction measurements were performed using a single-item measure, which is not as reliable or valid as multi-item measures but is widely used in research surveys and has been reviewed as acceptably valid [10,93]. Fourth, this study was conducted with health science students, who are more aware of the importance of health behaviors, including physical activity and diet; therefore, caution is needed when extrapolating the results to broader populations.

Although this study identified significant predictors of weight status, body, health and life satisfaction in a large sample of university students, the findings should be interpreted with caution due to the cross-sectional design. Longitudinal research is required in the future to verify the findings of the current study.

## 5. Conclusions

Our study provides valuable insights into the impact of lifestyle, symptoms of depression and eating behaviors on the weight status and body, health and life satisfaction of young adults, laying the groundwork for future research and interventions in this population.

The health behaviors developed in this period can persist into later life. Young adulthood is a key period for promoting healthy behaviors such as a healthy diet and a good level of physical activity, which, as shown herein, have a positive impact on body, health and life satisfaction. The lifestyle assessment of young adults should not focus on a single behavior; the co-occurrence and interaction of multiple health behaviors should be taken into consideration. It is also important to pay more attention to emotional eating and its negative consequences.

The importance of the high prevalence of depressive symptoms among young adults should also be highlighted. These symptoms were associated with reduced body, health and life satisfaction, while a history of depression significantly increased the risk of overweight/obesity in women. Screening for depression in young adults is necessary. Collaboration between specialists in obesity medicine, dietetics, psychology and psychiatry is needed for the treatment of people with obesity and depression.

Young adulthood is considered a critical life stage for interventions designed to improve health behaviors. We hope that the results of our study may contribute to the development of educational programs and intervention strategies.

## Figures and Tables

**Table 1:** Characteristics of the participants.

Characteristic	*N* (%)
Weight status	
Underweight	74 (9.3)
Normal range	574 (71.8)
Overweight	114 (14.3)
Obese	38 (4.8)
Symptoms of depression	
Normal range	401 (50.4)
Mild to moderate	255 (32.1)
Moderate to severe	104 (13.1)
Severe	35 (4.4)
History of depression	
Yes	52 (6.5)
No	748 (93.5)
Hypothyroidism	
Yes	68 (8.5)
No	732 (91.5)
Smoking	
Yes	157 (19.6)
No	643 (80.4)
Level of physical activity	
Low	164 (20.5)
Moderate	369 (46.1)
High	267 (33.4)
Body satisfaction	
Yes	498 (63.3)
No	289 (36.7)
Health satisfaction	
Yes	454 (57.3)
No	338 (42.7)
Life satisfaction	
Yes	607 (76.2)
No	190 (23.8)

**Table 2:** Participants’ body mass index, symptoms of depression, eating behaviors, diet, hypothyroidism, physical activity and body, health and life satisfaction broken down by gender.

	Men		Women		*p*
	Mean	SD	Mean	SD	
Body mass index	23.7	3.5	22.1	3.7	<0.001
Beck depression inventory	7.6	7.3	11.9	8.8	<0.001
Three-Factor Eating Questionnaire					
Cognitive restraint	39.5	18.6	44.2	20.3	0.010
Uncontrolled eating	36.1	18.9	33.5	18.0	n.s.
Emotional eating	28.0	25.6	39.7	27.3	<0.001
Food Frequency Questionnaire (aggregated)					
Sugar, sweets and snacks	1.4	1.1	1.6	1.2	n.s.
Milk, fermented milk drinks and curd cheese	1.2	0.9	1.1	0.8	n.s.
Sweetened milk products	0.4	0.5	0.3	0.4	0.008
Cheese	0.6	0.5	0.6	0.4	n.s.
Eggs and egg dishes	0.5	0.4	0.4	0.4	0.005
Breakfast cereals	0.2	0.3	0.2	0.3	n.s.
Whole-grain products	0.8	0.6	0.8	0.7	n.s.
Refined-grain products	1.0	0.6	0.8	0.5	<0.001
Butter and cream	0.8	0.6	0.6	0.6	0.023
Other animal fats	0.0	0.1	0.0	0.1	n.s.
Vegetable oils	0.5	0.4	0.5	0.4	n.s.
Other fats	0.4	0.5	0.2	0.4	0.003
Fruits	0.7	0.6	0.8	0.5	n.s.
Dried fruit, fruit preserves and fruit condiments	0.3	0.4	0.2	0.4	n.s.
Vegetables	0.9	0.5	0.9	0.6	n.s.
Dry and processed pulses	0.2	0.4	0.2	0.3	n.s.
Potatoes	0.5	0.4	0.4	0.3	0.004
Nuts and seeds	0.4	0.5	0.4	0.6	n.s.
Processed meats	0.9	0.8	0.5	0.6	<0.001
Red meat and venison	0.3	0.3	0.1	0.2	<0.001
White meat	0.4	0.3	0.3	0.3	<0.001
Fish	0.2	0.3	0.1	0.2	n.s.
Juices	0.5	0.6	0.4	0.5	0.004
Sweetened beverages and energy drinks	0.4	0.6	0.2	0.4	<0.001
Alcohol	0.4	0.4	0.2	0.3	<0.001
	*N*	%	*N*	%	*p*
Hypothyroidism					
Yes	3	2.0	65	10.0	0.003 ^Y^
No	146	98.0	586	90.0	
International Physical Activity Questionnaire					
Low activity	18	12.1	146	22.4	0.005
Moderate/high activity	131	87.9	505	77.6	
Smoking					
Yes	38	25.5	119	18.3	0.045
No	111	74.5	532	81.7	
Body satisfaction					
Yes	110	74.8	388	60.6	0.001
No	37	25.2	252	39.4	
Health satisfaction					
Yes	104	70.7	350	54.3	<0.001
No	43	29.3	295	45.7	
Life satisfaction					
Yes	125	84.5	482	74.3	0.009
No	23	15.5	167	25.7	

SD—standard deviation; n.s.—not significant; ^Y^ Yates’ correction.

**Table 3:** Multivariate logistic regression to predict overweight/obesity (separate models for all subjects, women, and men).

Variable	OR	95%CI	*p*
All participants			
Male gender	2.17	1.32–3.57	0.002
History of depression	1.95	0.98–3.87	n.s.
Cognitive restraint	1.01	1.00–1.02	0.019
Uncontrolled eating	0.99	0.98–1.01	n.s.
Emotional eating	1.02	1.01–1.03	<0.001
Refined grains	0.58	0.40–0.85	0.005
Red meat	3.15	1.40–7.08	0.005
Sweetened beverages	1.54	1.02–2.32	0.041
Alcohol	1.32	0.79–2.19	n.s.
Women			
Hypothyroidism	2.22	1.17–4.22	0.015
History of depression	2.24	1.07–4.67	0.032
Emotional eating	1.02	1.01–1.03	<0.001
Red meat	5.43	1.78–16.56	0.003
Men			
Cognitive restraint	1.03	1.01–1.05	0.011
Cereals	0.21	0.04–1.06	n.s.
Refined grains	0.73	0.35–1.52	n.s.
Oil	0.33	0.09–1.18	n.s.
Vegetables	0.45	0.18–1.09	n.s.

OR—odds ratio; CI—confidence interval; n.s.—not significant.

**Table 4:** Multivariate logistic regression to predict body satisfaction (separate models for all subjects, women, and men).

Variable	OR	95%CI	*p*
All Participants			
Male gender	1.56	0.94–2.58	n.s.
History of depression	0.63	0.31–1.27	n.s.
Overweight/obesity	0.33	0.21–0.51	<0.001
Symptoms of depression	0.16	0.11–0.23	<0.001
Cognitive restraint	0.98	0.97–0.99	<0.001
Uncontrolled eating	0.99	0.98–1.00	0.041
Emotional eating	1.00	0.99–1.01	n.s.
Moderate/high physical activity	2.08	1.36–3.19	0.001
Women			
History of depression	0.62	0.29–1.33	n.s.
Overweight/obesity	0.38	0.23–0.63	<0.001
Symptoms of depression	0.18	0.12–0.26	<0.001
Cognitive restraint	0.98	0.97–0.99	<0.001
Uncontrolled eating	0.99	0.97–1.00	0.024
Emotional eating	1.00	0.99–1.01	n.s.
Moderate/high physical activity	2.09	1.33–3.28	0.001
Men			
Overweight/obesity	0.14	0.05–0.39	<0.001
Symptoms of depression	0.08	0.03–0.23	<0.001
Emotional eating	1.00	0.98–1.02	n.s.
Moderate/high physical activity	1.82	0.51–6.47	n.s.

OR—odds ratio; CI—confidence interval; n.s.—not significant.

**Table 5:** Multivariate logistic regression to predict health satisfaction (separate models for all subjects, women, and men).

Variable	OR	95%CI	*p*
All participants			
Male gender	1.35	0.86–2.12	n.s.
Average financial status ^a^	1.52	0.86–2.69	n.s.
High financial status ^a^	1.88	0.92–3.83	n.s.
Hypothyroidism	0.23	0.13–0.44	<0.001
History of depression	0.19	0.08–0.44	<0.001
Overweight/obesity	0.60	0.40–0.91	0.016
Symptoms of depression	0.39	0.28–0.54	<0.001
Uncontrolled eating	1.00	0.99–1.01	n.s.
Emotional eating	0.99	0.98–1.00	0.004
Moderate/high physical activity	1.58	1.07–2.35	0.022
Women			
Average financial status ^a^	1.43	0.74–2.77	n.s.
High financial status ^a^	1.95	0.84–4.50	n.s.
Hypothyroidism	0.20	0.10–0.39	<0.001
History of depression	0.18	0.07–0.44	<0.001
Overweight/obesity	0.59	0.36–0.96	0.034
Symptoms of depression	0.39	0.27–0.56	<0.001
Uncontrolled eating	1.00	0.99–1.02	n.s.
Emotional eating	0.99	0.98–0.99	0.001
Moderate/high physical activity	1.65	1.08–2.52	0.022
Men			
Symptoms of depression	0.35	0.16–0.77	0.008
Uncontrolled eating	0.98	0.96–1.00	0.041

OR—odds ratio; CI—confidence interval; n.s.—not significant; ^a^ compared to low financial status.

**Table 6:** Multivariate logistic regression to predict life satisfaction (separate models for all subjects, women, and men).

Variable	OR	95%CI	*p*
All participants			
Male gender	1.17	0.67–2.04	n.s.
Average financial status ^a^	2.49	1.39–4.45	0.002
High financial status ^a^	8.67	3.43–21.95	<0.001
History of depression	0.55	0.28–1.08	n.s.
Smoking	0.60	0.38–0.94	0.027
Symptoms of depression	0.19	0.12–0.30	<0.001
Uncontrolled eating	0.99	0.98–1.01	n.s.
Emotional eating	1.00	0.99–1.00	n.s.
Moderate/high physical activity	1.70	1.11–2.61	0.015
Women			
Average financial status ^a^	2.95	1.53–5.67	0.001
High financial status ^a^	10.04	3.45–29.24	<0.001
History of depression	0.57	0.27–1.18	n.s.
Smoking	0.61	0.37–1.01	n.s.
Overweight/obesity	0.73	0.43–1.23	n.s.
Symptoms of depression	0.20	0.12–0.32	<0.001
Uncontrolled eating	0.99	0.98–1.01	n.s.
Emotional eating	1.00	0.99–1.01	n.s.
Moderate/high physical activity	1.66	1.05–2.63	0.030
Men			
Symptoms of depression	0.16	0.06–0.43	<0.001

OR—odds ratio; CI—confidence interval; n.s.—not significant; ^a^ compared to low financial status.

## Data Availability

The data presented in this study are available upon request from the corresponding author.
